# Comprehensive Review of Human *Plasmodium falciparum*-Specific CD8+ T Cell Epitopes

**DOI:** 10.3389/fimmu.2019.00397

**Published:** 2019-03-21

**Authors:** Janna Heide, Kerrie C. Vaughan, Alessandro Sette, Thomas Jacobs, Julian Schulze zur Wiesch

**Affiliations:** ^1^Infectious Diseases Unit, I. Department of Medicine, University Medical Center Hamburg-Eppendorf, Hamburg, Germany; ^2^German Center for Infection Research (DZIF), Partner Site Hamburg-Lübeck-Borstel-Riems, Hamburg, Germany; ^3^Division of Vaccine Discovery, La Jolla Institute for Immunology, La Jolla, CA, United States; ^4^Department of Medicine, Division of Infectious Diseases, University of California, San Diego (UCSD), La Jolla, CA, United States; ^5^Protozoa Immunology, Bernhard-Nocht-Institute of Tropical Medicine, Hamburg, Germany

**Keywords:** malaria, *Plasmodium falciparum*, CD8+, T cell epitope, HLA, restriction, cytotoxic T cells

## Abstract

Control of malaria is an important global health issue and there is still an urgent need for the development of an effective prophylactic vaccine. Multiple studies have provided strong evidence that *Plasmodium falciparum*-specific MHC class I-restricted CD8+ T cells are important for sterile protection against *Plasmodium falciparum* infection. Here, we present an interactive epitope map of all *P. falciparum*-specific CD8+ T cell epitopes published to date, based on a comprehensive data base (IEDB), and literature search. The majority of the described *P. falciparum*-specific CD8+ T cells were directed against the antigens CSP, TRAP, AMA1, and LSA1. Notably, most of the epitopes were discovered in vaccine trials conducted with malaria-naïve volunteers. Only few immunological studies of *P. falciparum-specific* CD8+ T cell epitopes detected in patients suffering from acute malaria or in people living in malaria endemic areas have been published. Further detailed immunological mappings of *P. falciparum*-specific epitopes of a broader range of *P*. *falciparum* proteins in different settings and with different disease status are needed to gain a more comprehensive understanding of the role of CD8+ T cell responses for protection, and to better guide vaccine design and to study their efficacy.

## Introduction

Malaria remains one of the pressing global health issues with ~216 million cases per year (1). The most virulent *Plasmodium* species, *Plasmodium falciparum (P. falciparum)*, accounts for the vast majority of the 445,000 deaths which occurred in 2016 due to malaria. Rising rates of drug (parasite-related) and insecticide (vector-related) resistance underscore the urgent need for an effective vaccine (2). The feasibility of achieving sterile protection by vaccination has already been demonstrated more than 50 years ago by experiments that could induce sterile immunity using irradiated sporozoites in mice (3) and in humans (4, 5). But the immunization of large numbers of individuals using sporozoites remains challenging and there is limited evidence for durable, high-level efficacy against natural exposure (6, 7). So far, the development of a subunit vaccine (that contains only the antigenic parts of a pathogen) has not succeeded despite decades of research (8, 9). In order to achieve sterile protection against *P. falciparum* infection, a better understanding of host-parasite interaction, and correlation of a protective *P. falciparum*-specific immune response is necessary. In particular, detailed knowledge of the *P. falciparum*-specific CD8+ T cell epitope repertoire is needed to further optimize vaccine design and to improve immune monitoring of future clinical malaria vaccine trials. In this review, we summarize and discuss all of the *P. falciparum*-specific CD8+ T cell epitopes in humans that have been identified so far, their potential significance as well as potential knowledge gaps.

## The RTS,S Vaccine and CSP-Specific CD8+ T Cells

There are currently several vaccine trials under way. However, recently published trials could only demonstrate suboptimal effectivity (10, 11). A malaria subunit vaccine, called the RTS,S vaccine (Mosquirix) targeting the CSP protein is the most advanced vaccine (12). Unfortunately vaccine efficacy was only modest (13), waned over time, and was not detectable any more three years after vaccination (11). Elevated IFNγ-levels after antigen-specific short-term cultivation and re-stimulation were detected by ELISPOT in subjects vaccinated with the RTS,S and CSP constructs and their levels decreased with waning protection (14). These studies demonstrate the importance of *P. falciparum*-specific T cells and the need to improve future strategies to induce a strong, broad and long-lasting vaccine-induced *P. falciparum*-specific CD8+ T cell response (15).

## Challenges of Malaria-Vaccine Development

Some of the particular obstacles of the development of a protective malaria vaccine are:

- The large size of the 23-megabase nuclear genome which consists of 14 chromosomes encoding for about 5,300 genes (16),
- the great genetic heterogeneity of some regions of those genes, and
- the complex life cycle and expression pattern of these genes (17),
- rendering malaria vaccine design more complicated than for most viral and bacterial infections.

A detailed understanding of the life cycle of the parasites and determination of the immune responses that confer protection after natural infection will provide important insights for future vaccine development. Within the human host, two main malaria life cycle stages are completed (18): the pre-erythrocytic stage and the blood stage. The pre-erythrocytic stage can be divided into an early sporozoite stage and the liver stage. During the early sporozoite stage, sporozoites are injected into the skin, enter the blood stream, and consecutively infect hepatocytes. This stage lasts minutes to hours (19). The ensuing liver stage consists of asexual replication and the maturation of sporozoites into schizonts within the hepatocytes, which lasts for ~1 week in humans in *P. falciparum* malaria (20). The subsequent blood stage is initiated with the release of merozoites that infect red blood cells (RBCs) and is the period in which clinical symptoms occur. These symptoms are partly induced by excessive host immune responses [reviewed in Artavanis-Tsakonas et al. (21)]. A small number of parasites in the blood develop into sexual-stage gametocytes, which can be taken up by the mosquito and continue the cycle of infection. The proteins that are expressed by plasmodia are life cycle stage specific: During the liver stage different (surface-) proteins like the circumsporozoite protein (CSP), liver stage antigens (LSA) and thrombospondin-related anonymous protein (TRAP) are expressed, while the merozoite surface protein (MSP) are expressed during the blood stage. The apical membrane antigen 1 (AMA1) is present in both stages (22). *P. falciparum*-specific T cells are primed within different immunological environments which might influence breadth and quality of the *P. falciparum*-specific response against the different antigens. It has been proposed that parasite-specific CD8+ T cells are mostly primed during the liver stage, while *P. falciparum*-specific CD4+ T cells enable clearance of the blood stage infection (23). CD8+ T cells are probably not important effector cells during the blood stage because the parasite-infected human erythrocytes do not display MHC class I molecules (24).

Nevertheless, naturally acquired immunity against malaria is primarily directed against blood stage antigens (mainly mediated via antibodies) and therefore prevents clinical disease but not the infection of hepatocytes (6, 7, 25), suggesting that there is no, or only little naturally acquired immunity against the pre-erythrocytic stage (26, 27). Of note, the pre-erythrocytic stage represents a bottleneck for the parasite (and is an ideal target for the host specific immune response) because of the relatively low number of parasites passing through this phase (~100 sporozoites, only) (28). A strong, specific-CD8+ T cell response directed against the liver stage would optimally prevent the parasite's transition to the blood stage and clinical symptoms and disease could be avoided (15).

## The *P. falciparum*-Specific CD8+ T Cell Response

Plasmodia, like other pathogens, induce a variety of immune effector responses: *P. falciparum*-specific antibodies, parasite-specific CD8+, and CD4+ T cells, as well as certain cytokines have been implicated as important effectors [reviewed in Stevenson and Riley (29) and Dobaño and Moncunill (30)]. The current review will focus on human *P. falciparum*-specific CD8+ T cells since there is strong experimental evidence in rodent and primate models as well as in humans that CD8+ T cells play a major role in providing protection:

- Sterile immunity using irradiated sporozoites in mice could be induced. This immunity was specific against the liver phase and later inoculation with parasitized red blood cells led to infection (3).
- Mice and rhesus monkeys were protected by immunization with attenuated sporozoites. This immunity was abrogated by experimental depletion of CD8+ T cells (31–34).
- The transfer of CSP-specific CD8+ T cell clones, as well as the transfer of a defined CD8+ T cell epitope conferred protection to malaria-naïve mice (35–39). Likewise, TRAP-peptides from *P. berghei* and a novel liver stage antigen MIF-4-like protein peptide Kb-17 have been able to elicit a CD8+ T cell-dependent response against murine malaria (40, 41).
- Immunity provided by antibodies was shown to be suboptimal most likely because blood-stage surface antigens show great variability (42, 43). Mouse models demonstrated that a robust CD4+ and CD8+ T cell response improves immunity since these T cell responses target internal antigens that are more likely to be conserved (44–46).
- Mouse models could show that induction of extremely high numbers of memory CD8+ T cells were a prerequisite for solid, sterile protection (47, 48).
- CD8+ T cells have shown to be important effectors that form clusters around infected hepatocytes and destroy them (49, 50). The importance of liver resident CD8+ T cells in protective immunity induced by attenuated *P. berghei* sporozoites has also been reported (51, 52). Mounting evidence suggests that effector CD8+ T cells eliminate the parasites without direct contact with infected hepatocytes via cytokine release (53). This model is also supported by the lymphogenic features of the liver (53).
- In humans, the inoculation with intact sporozoites (that were not attenuated) led to an increase of parasite-specific pluripotent effector memory T cells (54). The levels of *P. falciparum*-specific CD8+ T cells were also higher in those subjects who were protected after immunization with irradiated sporozoites compared to unprotected subjects (55).
- The polymorphism of CSP is primarily located in the region of identified CTL and T helper epitopes (56).
- An association with HLA class I and the course of the disease has been described showing that HLA-B^*^53, was associated with resistance to severe malaria (57). MHC class I-dependent presentation of antigens in *P. berghei* malaria was also demonstrated in the mouse model (58).
- Humans immunized with irradiated sporozoites or naturally exposed to malaria can generate a CTL response to pre-erythrocytic-stage antigens (8, 59–63).
- In mouse models, an excessively strong CD8+ T cell response has been associated with the development of cerebral malaria (64), and a deeper understanding of the CD8+ T cell repertoire may have implications beyond vaccine development and could be relevant for the clinical course of this disease in humans.

The mechanism of protection by CD8+ T cells is thought to be partly cytokine-mediated by interferon-γ (IFNγ) (31) and tumor necrosis factor (TNF) [reviewed in Dobaño and Moncunill (30)] that both inhibit parasite development. Perforin and granzymes kill infected hepatocytes through direct lysis (39), (65–68). IFNγ responses directed against pre-erythrocytic stage antigens of *P. falciparum* are associated with higher hemoglobin levels and significantly reduced prevalence of severe anemia (69) and are associated with significant resistance to re-infection (70, 71). It could also be shown that age-related cumulative exposure to *P. falciparum* increases the frequency of IFNγ responses (72, 73). This demonstrates the importance of IFNγ production for protection against malaria.

## Challenges of Investigating the *P. falciparum*-Specific CD8+ T Cell Response

So far, the breadth and specificity of the parasite-specific CD8+ T cell response in malaria has only been poorly described. One problem of the mapping of *P. falciparum*-specific T cell responses is the comparatively low *ex vivo* frequency of circulating peripheral *P. falciparum*-specific T cells (74, 75). There are several proposed hypotheses to explain low frequencies and responses in peripheral blood samples:

- Lymphopenia occurs during acute malaria infection [reviewed in Scholzen and Sauerwein (76)]. T lymphocytes are thought to migrate into different tissues, e.g., the liver (8, 77).
- There is a high genetic diversity between different *P. falciparum* strains with high mutation rates of several antigenic regions of the genome that complicates detection of T cells (Supplementary Figures 1–4). Additionally, no optimized consensus sequence for screening purposes of T cell responses against malaria proteins has been designed as of now (78–80). This extreme genetic diversity, particularly of the surface antigens, makes the detection of *P. falciparum*-specific T cells difficult (78–80).
- The immunoregulatory effects of a blood stage infection are thought to influence the priming of an adaptive immunity against the pre-erythrocytic stage (81–83).
- *P. falciparum* contains thousands of antigens that could serve as potential T cell epitopes. This sheer breadth of epitopes primed during an acute infection could also explain the low frequencies of specific T cells against an individual T cell epitope (84).
- The inability to generate new responses against emerging variants (original antigenic sin), is a phenomenon that has been described for several pathogens like influenza, HIV, and the hepatitis B virus (85–91). Malaria can be caused by infection with multiple *Plasmodium* strains (92, 93) and limited responsiveness of CD8+ T cells in coinfection with different *Plasmodium* strains could be shown (94) similar to the limited cross-genotype responsiveness after infection with different HCV genotypes (95).
- The human immune T cell response is modulated and dampened by the parasite through several mechanisms (83, 96). Walther et al. showed that regulatory T cells were rapidly induced following blood-stage infection and were associated with a burst of TGF-β production, decreased proinflammatory cytokine production, and decreased antigen-specific immune responses (96). Mackroth et al. demonstrated that acute *P. falciparum* malaria induced *P. falciparum*-specific PD1+CTLA4+CD4+ T effector cells that co-produced IFNγ and IL-10 and inhibited other CD4+ T cells (83). In rodent malaria it could be shown that PD-1 mediates up to 95% reduction in numbers and functional capacity of parasite-specific CD8+ T cells (97).
- Evasion mechanisms like Kupffer cell apoptosis and reduced expression of major histocompatibility complex (MHC)-I also resulted in T cell tolerance (98). Additionally, a reduced APC (antigen presenting cell) function of the Kupffer cells from sporozoite-infected mice was shown which might also explain the comparatively low magnitude of *P. falciparum*-specific T cell responses detected in malaria patients (99).

## The Role of HLA Alleles in Malaria

Certain human leukocyte antigens (HLAs) like HLA-B53, DRB^*^13:02, and DQB^*^05:01 are associated with protection from severe malaria (84, 100). This suggests that selection of particular epitopes might be associated with better control of parasitaemia. Gabonese children carrying the HLA class II allele DQB1^*^0501 had a higher frequency of IFNγ-responses to LSA1 T cell epitopes, compared with non-carriers, and were better protected against malaria anemia and re-infections (100).

**APFISAVAA** (LSA3), **EPKDEIVEV** (LSA3), and **KPIVQYDNF** (LSA1) are the only known CD8+ T cell epitopes with HLA-B53 restriction and are located on the liver stage antigen 1 (LSA1) and the liver stage antigen 3 (LSA3) (23, 63, 100–103).

The genes coding the HLA molecules are the most polymorphic genes in humans and several observations indicate that this is due to selective immunological pressure (84). This polymorphism leads to varying degrees of protection against infectious pathogens (but also to association between MHC types and autoimmune diseases) (104). *P. falciparum* infection has been a major selective force in human evolution, especially in West Africa, where the HLA alleles HLA-A^*^53, DRB1^*^13:02, and DQB1^*^05:01 are more frequent than in the rest of the world (allelefrequencies.net) (105). But *P. falciparum* contains thousands of different potential T cell epitopes and multiple immune responses are evoked by a single infection (74). This implies that most of these responses are possibly of limited protective value and only a few epitopes will elicit strong, long lasting and protective immune answers (84). However, longitudinal studies about the relationship between HLA alleles, cytokine patterns, and the outcome of *P. falciparum* infection are rare (100) and other protective HLA alleles have not yet been identified in humans.

## Methods for Detection of Antigen-Specific T Cells

Over the last 20 years, the methodologies of T cell detection have undergone considerable changes. *P. falciparum*-specific T cells have been detected by a number of different assays that differ by practicability, price, sensitivity, and functional read-out. Different assays have different advantages and disadvantages, which has to be taken into account when comparing results of different studies that have characterized *P. falciparum*-specific T cell responses over the last decades. The most commonly used techniques for T cell analysis in malaria are shown in Table 1.

**Table 1:** Different methods for detection of *P. falciparum*-specific CD8+ T cells.

**Method**	**Relevance for T cell detection in malaria**	**Advantages and limitations**
Chromium-51 (51Cr) release assay	• Used for CD8+ T cell detection in malaria from 1991 to 2004 and the majority of published malaria-specific T cell epitopes were discovered by this method	• Specific for cytolysis • Sensitive (1 cell out of 100) • Specific infrastructure required (107) • Expensive, work, and time intensive
ELISA	• Few studies have used ELISA for detection of malaria-specific CD8+ T cell epitopes to date (70, 74, 100, 108)	• 400-fold less sensitive than ELISPOT • Analyzes cytokines and not direct cytolysis (109)
[3H]-thymidine incorporation assay	• Commonly used in cell proliferation assays but mostly for CD4+ T cell detection (110) • Only one study detected a CD8+ T cell epitope shorter than 20 AA (69)	• Error prone, unspecific at times • No info on *ex vivo* phenotype of T cell • Specific infrastructure required (111)
ELISPOT	• Sensitive and robust immunological method for enumerating antigen-specific lymphocytes (112, 113) • Not used for specific T cell detection in the field of malaria until 1999 (114)	• Sensitive, cost effective technology, easy to set up in tropical regions • CD4+ T cell depletion is necessary to link cytokine production to CD8+ T cells (115) • No information on *ex vivo* T cell phenotype
ICS	• Only two studies have used ICS (intracellular cytokine staining) technology for Plasmodium-specific CD8+ T cell mapping (116, 117)	• Cell, cost, and work intensive assay (118)
MHC class I-multimer	• Only two studies have performed MHC multimer staining of vaccine-induced *Plasmodium*-specific CD8+ T cell responses (119, 120)	• Sensitive and specific technology • Detection is uncoupled from function and thus T cells can be detected that do not produce cytokines at the time of examination (118) • HLA molecules of patients need to be determined • Expensive and error prone technology, few *P. falciparum*-specific MHC multimers have been established so far

Potential difficulties of the generally low frequencies of the *P. falciparum*-specific T cells can be overcome by *in vitro* expansion and short-term culture methods. Numerous studies have cultivated PBMCs with the particular antigen for a few days up to 2 weeks to allow expansion of the specific T cells (74, 121). However, *in vitro* expansion alters the T cell function and phenotype to some extent. It is also difficult to extrapolate the original specific *ex vivo* T cell frequency after culture. An alternative approach to increase low frequencies of antigen-specific T cells is the quantitative pre-enrichment of target cells via magnetic cell separation (118). This approach allows phenotypic assessment of the specific T cells but has not yet been used in the context of malaria CD8+ T cell research.

## Resources for *P. falciparum*-Specific Epitope Research and *in silico* Analysis

To date, there are several online *P. falciparum*-specific resources available on the internet that provide immunological information and/or data related to plasmodial antigens, or that offered links to clinical trial data. This section provides a brief overview of these important resources.

## The IEDB: An Online Epitope Resource

The Immune Epitope Database and Analysis Resource (IEDB.org) was created by the National Institute of Allergy and Infectious Disease (NIAID) to provide the scientific community with a repository of freely accessible immune epitope data (122, 123). The IEDB contains data captured from the published literature, as well as data submitted through NIAID's high-throughput epitope discovery efforts. The IEDB includes antibody and T cell data from human, non-human primate, and rodent hosts, as well as other animal species, and encompasses epitopes associated with all infectious diseases, allergy, autoimmunity and transplantation and/or alloreactivity. In addition to antibody and T cell response data, the IEDB also contains MHC binding and MHC ligand elution data (mass spectrometry).

It thus provides a unique resource to inventory and analyze immunological data for a given pathogen or disease and has been utilized to produce several meta analyses including a 2009 analysis of epitopes derived from *Plasmodium* (110).

Currently, the IEDB contains 3,000 unique epitopes derived from the genus *Plasmodium*. Of these data, species causing malaria, *P. falciparum, P. vivax*, and *P. malariae*, represent 88% of the total, with *P. falciparum*-specific determinants most prominent among them (2,332). *P. falciparum*-specific data include epitopes defined for more than 160 antigens, including both protein and non-protein (cell surface glycolipids) targets. Data related to human disease are most numerous, with 1,485 epitopes defined from 126 different plasmodial antigens and reported from 295 references. These were data derived from more than 3,900 immunoassays (2,205 T cell and 1,693 antibody). Data generated using murine models of disease (710 epitopes) and non-human primates (104 epitopes) are also available. At the level of immune response phenotype, there are currently 774 human antibody epitopes from 120 antigens and 843 T cell epitopes from 32 antigens, including linear and non-linear antibody epitopes, and class I (147) and class II (596). Of the 147 MHC class I/ CD8+ T cell epitopes, we included 132 epitopes in this review. Fifteen epitopes were excluded because they were longer than 20 amino acids (AA).

In addition to its epitope data repository, the IEDB hosts an array of epitope prediction and analysis tools, including those for T cell class I and II prediction (tools.iedb.org/main), such as TepiTool (124), the T cell class I pMHC immunogenicity predictor (125), and the CD4+ T cell immunogenicity prediction tool (126). Useful among the other analysis resources are the Immunome Browser, which maps epitope prominence along the antigen, the Epitope Cluster Analysis Tool 2.0, and the Epitope Conservation Analysis tool (127).

## Other *P. falciparum*-Specific Online Resources

Informatic sites containing plasmodial antigen sequence and/or structural data include the *Plasmodium* Genomics Resource (plasmodb.org/plasmo) (128) and the *Plasmodium* vivax Structural Databank (PvaxDB) (scfbio-iitd.res.in/PvaxDB) (129). The Rodent Malaria genetically modified Parasites (RMgmDB) (pberghei.eu/index.php) site (130) contains genetically modified rodent malaria parasites that have been generated by many labs worldwide. The Malaria Data site (ebi.ac.uk/chembl/malaria) provides a searchable and downloadable public resource for targets, compounds, assays, and other data related to malaria research (131). Similarly, the Malaria Immunology database (malarimdb.org/) provides users an overview of the roles of the various immunological, metabolic vascular, and erythrocytic factors involved in blood-stage malaria and includes human patient data, as well as data from murine models of disease (132). The Malaria Atlas Project (map.ox.ac.uk) enables users to download, visualize and manipulate global parasite rate survey data (133). MalariaGen (malariagen.net) is a genomic epidemiology network for next generation DNA sequencing tools and technologies (134).

## Comprehensive Review of *P. falciparum*-Specific CD8+ T Cell Responses

**Figure 1:**
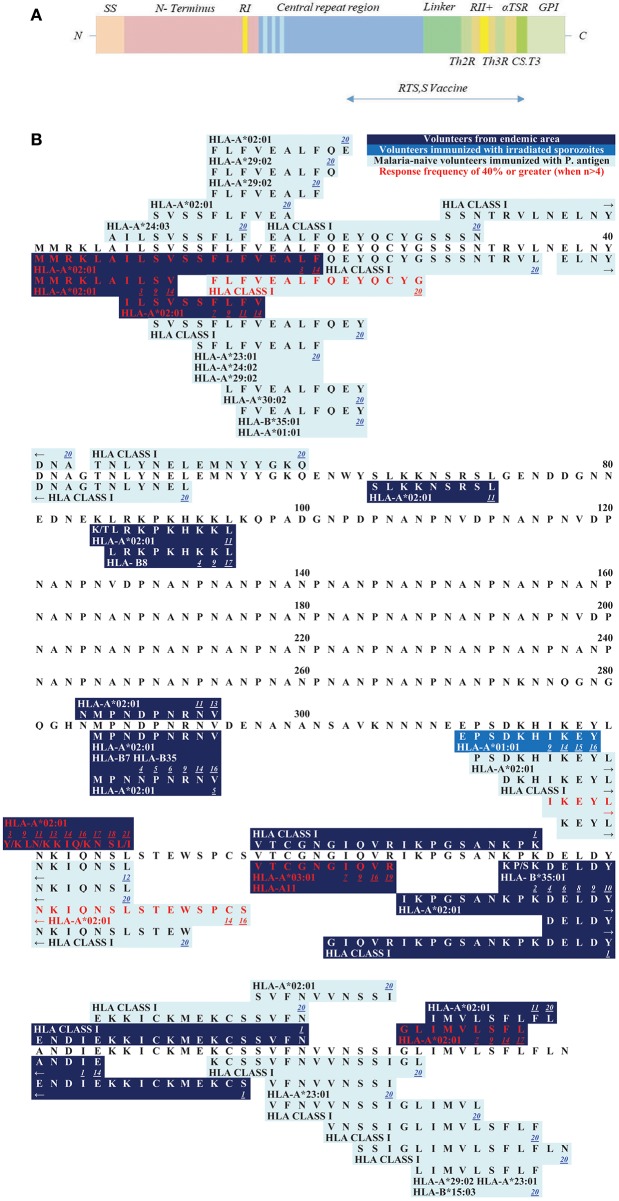
**(A)** Schematic draft of *P. falciparum* CSP. The signal sequence (SS) and region one (RI) are localized within the N-terminus. The central repeat region is a region of NANP (dark blue) and NVDP (light blue) repeats. The C-terminus (green shades) is linked to the repeat region and contains an α1-helix thrombospondin type-1 repeat (αTSR). The αTSR contains the overlapping Th2-Region (Th2R), region II+ (RII+), Th3- Region (Th3R), and CS.T3. The glycosylphosphatidylinositol (GPI) attachment site is an anchor for the protein to the sporozoite plasma membrane (135, 136). The RTS,S vaccine contains a part of the central repeat region and the C-terminus. **(B)** Epitope map of CSP. All MHC class I epitopes that can be found for the CSP (circumsporozoite protein) are marked in this epitope map (epitopes longer than 20 AA were excluded). Dark blue boxes: naturally exposed volunteers; blue boxes: volunteers who were immunized with attenuated sporozoites; light blue boxes: malaria-naïve volunteers immunized with *P. falciparum* antigens. Red font color: response frequency of 40% or greater (when more than 4 subjects were tested). The small number next to the epitope is a link to the reference that published the epitope (you will be forwarded to the journal website by clicking onto the link). Within each box the according MHC type is marked and variants within the epitope sequence are indicated by a dash. Most epitopes that were detected within the N-terminus of CSP were found by Sedegah et al. (137). Within the central tandem repeat region no CD8+ T cell epitopes have been found so far. The C-terminus is a very immunogenic region of this protein and contains most of the epitopes.

**Figure 2:**
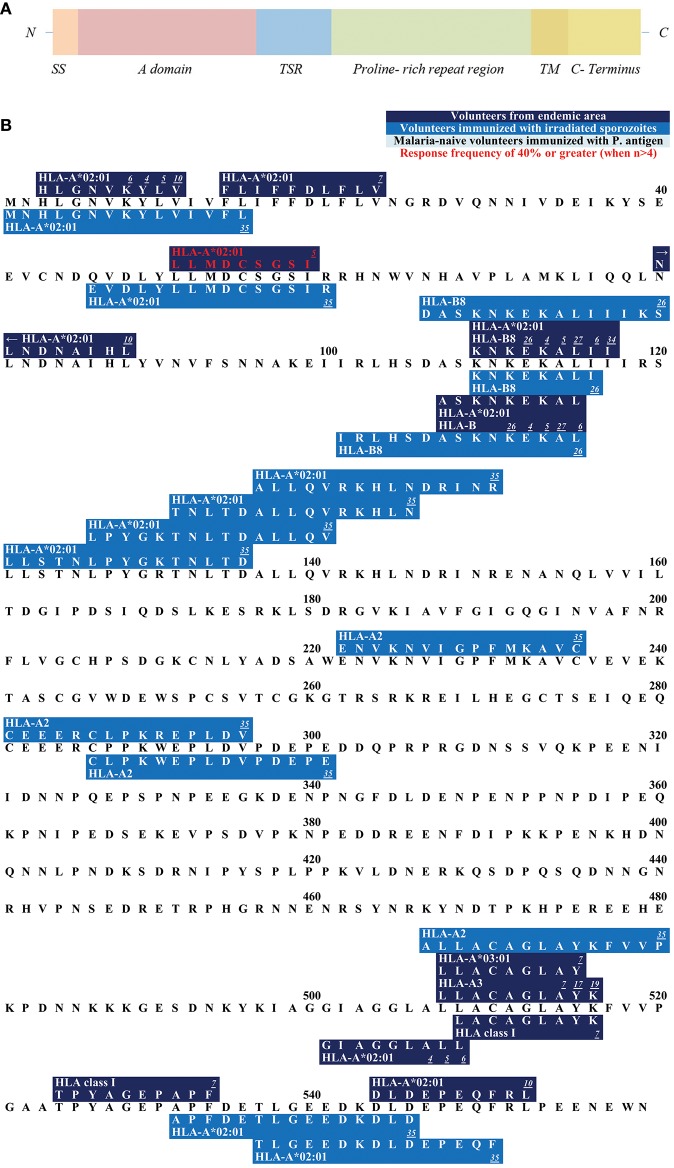
**(A)** Schematic draft of *P. falciparum* TRAP. This schematic draft shows the signal sequence (SS) at the N- terminus containing two adhesive domains i.e., the A domain (von Willebrand factor A- like domain) and the TSR (thrombospondin type-I repeat) domain (138, 139). This is followed by a proline-rich repeat region, a transmembrane domain (TM) and finally the acidic C-terminus located in the cytosol (140). **(B)** Epitope map of TRAP. Most epitopes within TRAP have been detected in studies that worked with malaria-naïve volunteers who were immunized with attenuated sporozoites.

**Figure 3:**
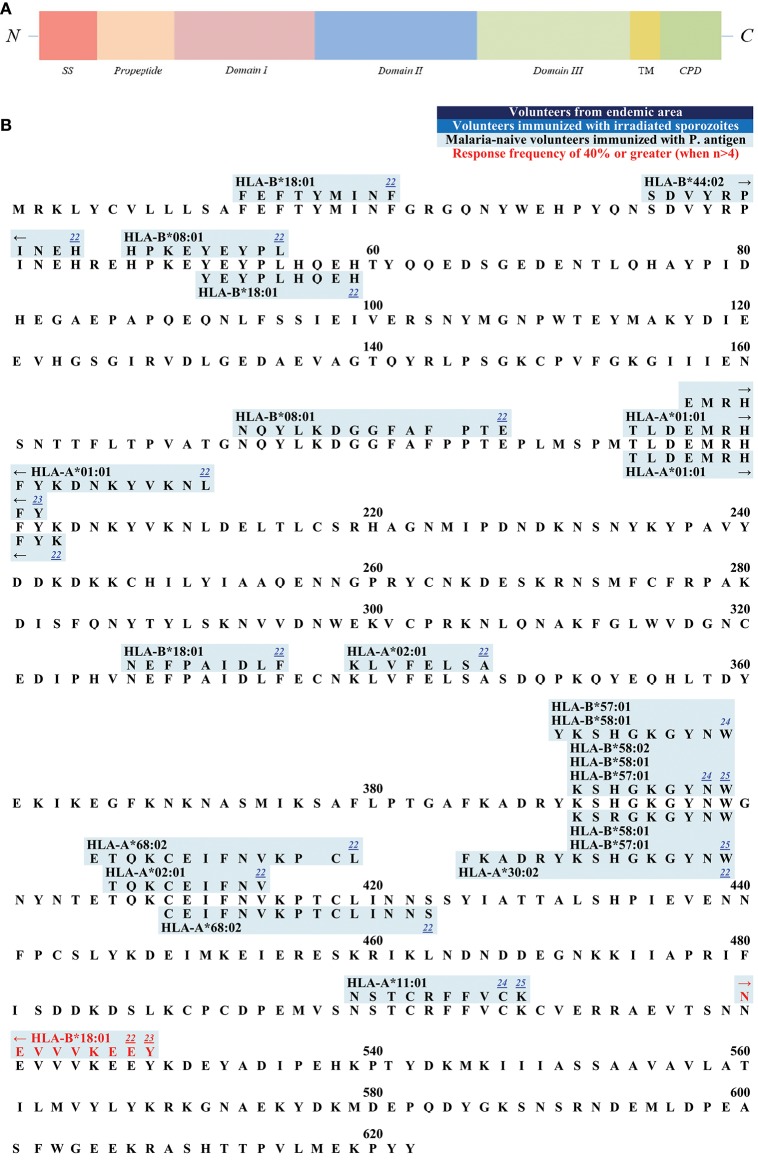
**(A)** Schematic draft of *P. falciparum* AMA1. This schematic draft shows the signal sequence (SS) toward the N- terminus followed by the propeptide which is cleaved off during the maturing process. Domain I, II, and III build the ectodomain of AMA1 with a hydrophobic cleft and a PAN fold in the tertiary structure. The transmembrane domain (TM) as well as the cytoplasmic domain (CPD) are located within the C-terminus. **(B)** Epitope map of AMA1. Only epitopes that were tested in malaria-naïve study cohorts immunized with *P. falciparum* antigens have been published for AMA1. NEVVKEEY is the only epitope within this antigen that reached a response RF (response frequency) of 40% or greater.

**Figure 4:**
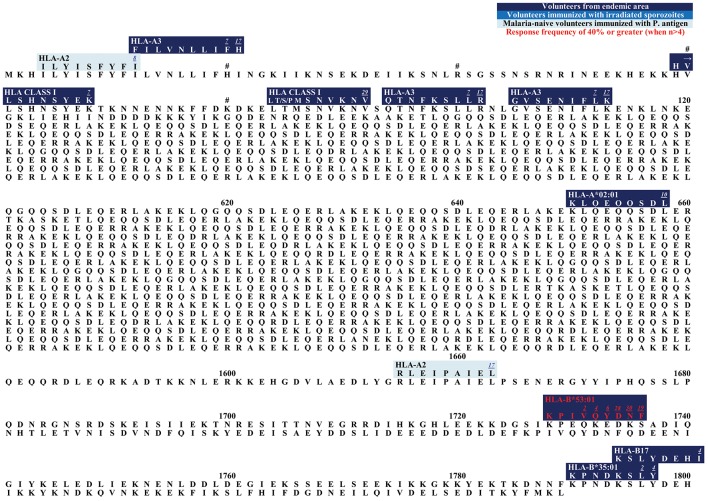
Epitope map of LSA1.

**Figure 5:**
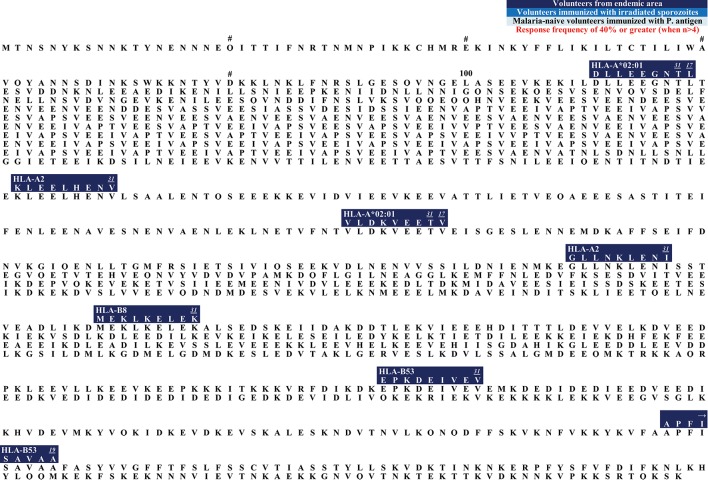
Epitope map of LSA3.

**Figure 6:**
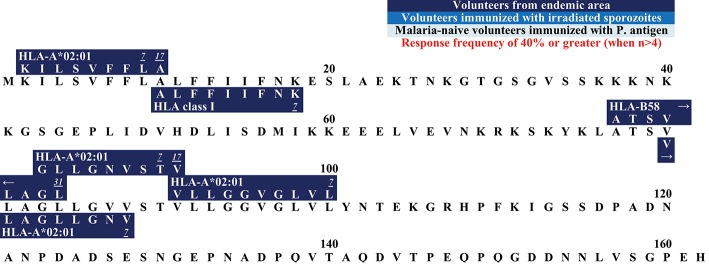
Epitope map of EXP1.

**Figure 7:**
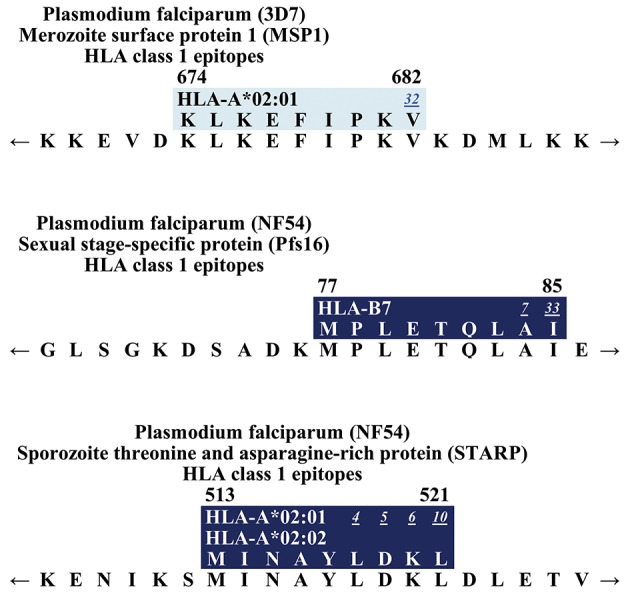
Epitope map of MSP1, Pfs16, and STARP.

While a great number of *P. falciparum*-specific T cell responses can be searched in the IEDB (122), a comprehensive review providing an overview of discovered CD8+ T cell epitopes in *P. falciparum* and discussing them in context is lacking, to date. Therefore, we conducted an exhaustive database (www.iedb.org) (Terms: *P. falciparum*; MHC Class I; Humans) and literature review (PubMed)^1^ (Terms: *P.falciparum*; CD8+ T cells; epitopes; cytotoxic T cells; MHC class I) and summarized our findings in form of an epitope map (Figures 1–**7**). We defined an epitope as a *P. falciparum*-specific peptide of 20 AA (amino acids) or less that elicited a CD8+ T cell response. Fifteen epitopes that had shown to be restricted to MHC class I, but were longer than 20 AA, were excluded from our review (106, 116). Epitopes described for other *Plasmodium* species or T cell responses primed and detected in murine malaria infection were similarly not included in the current overview. Likewise, *P. falciparum*-specific CD4+ T cell responses were also not listed. The relative pattern of immune dominance or relative breadth and quality (e.g., cytokine pattern) of the response measured against each of the epitopes was also not assessed.

From the 147 CD8+ T cell epitopes found in the IEDB, we included 132. These epitopes were described in 36 different publications. Additional information about the studies, like the methods that were used, the experimentally assessed HLA restriction (if available) and the study population that was tested, was included for each epitope in Supplementary Table 1. We distinguished between four different types of study populations that were tested for specific CD8+ T cells:

1. Malaria-naïve volunteers immunized with *P. falciparum* antigens in a vaccine trial
2. Malaria-naïve volunteers immunized with attenuated sporozoites
3. Volunteers from a malaria endemic area naturally exposed to *P. falciparum*
4. Volunteers with known *P. falciparum* infection (acute or post clinical malaria).

The epitopes are then discussed in the context of the protein structure and their role in pathogenesis, as well as in terms of overall reported immunodominance. For this, response frequency (RF) data was used. RF represents the number of respondents over the number of subjects tested, when provided by the investigator for the respective assay. It has to be taken into account that some studies only included volunteers that carry the MHC allele HLA-A^*^02 (141). In this case the RF is not representative for a MHC diverse population.

The assessment of the breadth and specificity of a *P. falciparum*-specific immune response will be a useful tool for the field and provide better knowledge of the *P. falciparum*-specific T cell response on an epitope level. This will likely be crucial for the understanding of the exact role of CD8+ T cells for control of disease, for vaccine design and for monitoring of the CD8+ T cell response in future vaccine trials (142). Of the more than 5,000 *P. falciparum* proteins, *P. falciparum*-specific CD8+ T cell epitopes have only been described thus far for the following nine antigens:

1. Circumsporozoite protein (CSP)
2. Thrombospondin-related anonymous protein (TRAP)
3. Apical membrane antigen 1 (AMA1)
4. Liver stage antigen 1 (LSA1)
5. Liver stage antigen 3 (LSA3)
6. Circumsporozoite-related antigen (EXP1)
7. Merozoite surface protein 1 (MSP1)
8. Sexual stage specific protein 16 (Pfs16)
9. Sporozoite threonine and asparagine-rich protein (STARP).

The majority of published MHC class I restricted CD8+ T cell epitopes was detected on CSP, with 55 known epitopes. This corresponds to 42% of all CD8+ T cell epitopes. This is followed by TRAP with 28 CD8+ T cell epitopes (21%). Nineteen epitopes on AMA1, 13 epitopes on LSA1, 7 epitopes on LSA3, and 6 epitopes on EXP1 that are specific for MHC class I/ CD8+ T cells had been detected. For the antigens MSP1, Pfs16, and STARP only one CD8+ T cell epitope, respectively, has been found (63, 69, 74, 143).

The average response frequency of all assays combined (when provided) was 23% [2.2–100% (74)] (total number of assays: 228). An average of 2.4 [1–11 (144)] subjects responded and the average size of a study population was 10.3 subjects [1–45 (74)]. Looking only at the studies that included malaria-naïve volunteers (total number of assays: 97), an average of 26.8% [5.3–100% (145)] responded in an average group of 8.2 [1–19 (145)] subjects. The study cohorts with volunteers from a malaria endemic area had an average response frequency of 16.0% [2.2–100% (74)] (total number of assays: 91). The average size of these cohorts was 15.4 study subjects [1–45 (74)]. The study group of volunteers immunized with attenuated sporozoites was a lot smaller with an average of 3 [1–4 (146)] subjects tested. The average RF was 72.6% [50–100% (147)] (total number of assays: 40).

In the following section MHC class I-restricted*, P. falciparum*-specific CD8+ T cell epitopes that were published at least in three different publications and/or showed a response frequency of 40% or greater or other important features will be listed according to each of these proteins. Supplementary Table 1, which gives a more comprehensive overview, lists every CD8+ T cell epitope that has been published so far.

## Circumsporozoite Protein (CSP)

The circumsporozoite protein (CSP) is an important protein of the pre-erythrocytic stage of the parasite. It is the most abundant surface protein on sporozoites and has important roles in the *Plasmodium* life cycle (148). The function of CSP has been investigated in detail in *P. berghei* infection where it is essential for sporulation, gliding mobility and binding to hepatocytes (149–151). Via CSP, the sporozoites recognize heparan sulfate proteoglycans (HSPGs) which are glycoproteins on the surface of hepatocytes that allow cell interactions (152). The function of CSP in *P. falciparum* malaria is less well studied, but binding to hepatocytes has also been demonstrated (152).

The CSP protein is 397 amino acids in length and shows a similar structure among different *Plasmodium* species, suggesting conserved essential roles for each domain. The protein consists of the N-terminus, the central tandem repeat region and the C-terminus (Figure 1A) (153).

The N-terminus, and in particular the first 78 amino acids (AA), is a very well conserved part of the CSP (Supplementary Figure 1). It contains region I (RI), a five AA sequence (**KLKQP**) conserved in almost all *Plasmodium* species (154). The N-terminus is important for binding to hepatocytes (152, 155). Cleavage occurs at RI by a parasite protease upon contact with host hepatocytes and leads to a change of an “adhesive” to an “invasive” behavior (156, 157). Antibodies to RI can inhibit cleavage of CSP and therefore impair invasion *in vitro*, as well as *in vivo* (158).

The following epitopes localized within the N-terminus have been described to date (Figure 1B): The epitope **MMRKLAILSV** shows a high response frequency (RF) of 40% (6/15) in a study with volunteers from a malaria endemic area (159). The recognition of the peptide was demonstrated via an IFNγ-specific ELISA. It was also recognized by malaria-naïve volunteers immunized with a plasmid DNA vaccine (121, 144). 35.7% (5/14) to 36.4% (4/11) of the volunteers recognized the epitope. The overlapping epitope **MMRKLAILSVSSFLFVEALF** also had a high RF of 78.6% (11/14) in immunized but malaria-naïve volunteers (144, 159). The overlapping epitope **ILSVSSFLFV** showed response frequencies of 50% (1/2) to 71.4% (10/14) in studies with malaria-naïve volunteers who were immunized with CSP, but the epitope could also be detected in inhabitants from a malaria endemic-area [RF: 5.9% (1/17) to 8.3% (3/36)] (74, 121, 141, 144).

Within the N-terminus of CSP Sedegah et al. detected responses directed against 15 CD8+ T cell epitopes in 2013 (137). They performed an IFNγ-specific ELISPOT after adenovirus vector-based immunization of malaria-naïve volunteers with CSP and AMA1. Six to ten subjects were tested, and RF varied from 10% (1/10) to 40% (4/10).

The epitope **LRKPKHKKL** was detected in both, vaccinated malaria-naïve volunteers and volunteers from an endemic area and was restricted to HLA-B8 (63, 121, 145). **LRKPKHKKL** showed a RF of 18.2% (2/11) in a malaria-endemic study population (63).

The tandem repeat region located in the center of the protein is an immunodominant B cell region and most of the described *P. falciparum*-specific antibody-epitopes are located here (160, 161) (Figure 1A). This region comprises of the tetra peptide repeats NANP and NVDP. Notably, no *P. falciparum*-specific CD8+ T cell epitopes have been detected in this region, but several CD4+ T cell epitopes of different length have been found in this area (162–166).

Finally, the C-terminus contains a linker followed by an α-thrombospondin type-1 repeat (αTSR) domain (153, 167) (Figure 1A). Within the αTSR domain the region II-plus is located. This is an 18-amino acid sequence that also mediates adhesion of sporozoites to the heparan sulfate proteoglycans (HSPGs) (168, 169). CSP is anchored to the sporozoite plasma membrane by a glycosylphosphatidylinositol (GPI) attachment site at its C-terminus (170). Lymphoproliferative assays in naturally exposed patients have shown three immunodominant T cell epitope domains located within the C-terminus, called Th2R, Th3R, and CS.T3 (171).

Within the C-terminus of the protein, the following epitopes have been described (Figure 1B):

The epitope **MPNDPNRNV** was tested in malaria-naïve volunteers as well as in subjects from a malaria-endemic area. RF varied from 1/10 (10%) to 1/3 (33%) in malaria-naïve volunteers immunized with CSP (106, 121, 144, 172). The peptide is restricted by different HLA molecules: HLA-A^*^02:01, HLA-B7, and HLA-B35. Of note, HLA-B7 and HLA-B35 both belong to the HLA-B7 supertype (173). In volunteers from a malaria-endemic area 16.7% (1/6) to 100% (1/1) recognized the peptide (60, 63, 101).

Two epitopes are located within the Th2R region: **EPSDKHIKEY**, restricted to HLA-A^*^01:01 was only tested in malaria-naïve volunteers who were immunized with plasmid DNA or irradiated sporozoites (106, 121, 144, 174). RF varied from 10% (1/10) to 100% (2/2).

Wang et al. detected the epitope **IKEYLNKIQNSLSTEWSPCS** and RF was 35.7% (5/14) to 100% (8/8) (106, 144). The peptide **Y/KLN/KIQ/KNSL/I** is the best characterized T cell epitope and was described in 8 publications (106, 108, 119, 121, 141, 144, 145, 159, 175). It is also the most polymorph epitope within CSP (Supplementary Table 1). **YLNKIQNSL** was recognized in 23.5% (4/17) volunteers from a malaria-endemic area. In malaria-naïve volunteers who were immunized with a subunit vaccine RF was 13.3 (2/15) to 100% (5/5). **YLKKIKNSL, YLKKIQNSL**, and **KLKKIKNSI** were all detected in a malaria-endemic study population. RF for **YLKKIKNSL** was 60% (9/15) to 100% (1/1), for **YLKKIQNSL** 33.3% (5/15) and to **KLKKIKNSI** responded 41.2% (7/17) of the study population. The epitope **VTCGNGIQVR** is located within the region II-plus. This epitope was tested in subunit vaccine trials [RF = 10% (1/10) to 57.1% (4/7)], with irradiated sporozoites [RF = 100% (4/4)] and naturally exposed volunteers [RF = 17.8% (8/45) to 41.7% (5/12)] (74, 102, 121, 144). The epitope is restricted to HLA-A^*^03:01 and HLA-A^*^11 (121).

**KPKDELDY**, restricted to HLA-B^*^35:01 was detected in malaria-naïve [RF = 100% (1/1)] and naturally exposed subjects (RF = 12.5% (1/8) to 20% (3/15)] (23, 63, 94, 101, 121, 176). **KSKDELDY** had a RF of 10% (1/10) to 12.5% (1/8) in a naturally exposed population.

**GLIMVLSFL** was tested in subunit vaccine trials [RF = 6.7% (1/15) to 78.6% (11/14)], with irradiated sporozoites [RF = 100% (2/2)] and naturally exposed volunteers [RF = 9.1% (1/11) to 11.1% (4/36)] (74, 121, 145).

The CSP is the most frequently investigated antigen in terms of the number of different studies and number of described T cell epitopes. This is probably due to the fact that CSP was the first of the malarial proteins to be cloned, and as a result, significant efforts have been undertaken to investigate this protein (177). Ever since, CSP has been used as antigen for several vaccine trials so far (12, 178). The RTS,S vaccine contains half of the central repeat region and the entire C-terminus of CSP (Figure 1A) (12).

## Thrombospondin-Related Anonymous Protein (TRAP)

TRAP is a 559 AA long protein that induces antibody and T cell responses (179). This transmembrane protein is localized in the micronemes (specialized secretory organelles) and on the surface of sporozoites (180). It is important in the infection of hepatocytes (181, 182), has a function during the invasion of mosquito salivary glands, and TRAP also plays a role in sporozoite motility (140).

The N-terminus of TRAP contains the A domain and thrombospondin type-I repeat (TSR) which are two adhesive areas that can bind to heparan sulfate proteoglycans (HSPG) (181, 182) (Figure 2A). Upon contact with the host cell, TRAP is released from the micronemes and allows these adhesive domains to interact with the hepatocytes (181–184). Presumably, other non-HSPG-receptors also play a role for hepatocyte invasion (181).

The **C-terminus** of TRAP remains intracellularly located and is attached to the sporozoite motor system (169) (Figure 2A). The N-terminus and the intracellular C-terminus form a moving junction and allow gliding without cilia or flagella or major changes in the cell morphology (185, 186).

The N-terminus of TRAP contains 11 different epitopes (Figure 2B). **HLGNVKYLV** was tested in HLA-A^*^02:01 positive study participants who were naturally exposed to malaria. RF varied from 5.9% (1/17) to 100% (1/1). 11.1% (4/36) of volunteers from a malaria-endemic area and 50% (1/2) of volunteers immunized by irradiated sporozoites responded to the peptide (60, 63, 141).

**LLMDCSGSI** has shown to be restricted to HLA-A^*^02:01 and 44.4% (4/9) of volunteers naturally exposed to malaria responded to this epitope (60).

**ASKNKEKAL** was restricted by HLA-A^*^02:01 and HLA-B (60, 63, 101, 147, 187) and was recognized by 9.1% (11/1) to 33.3% (2/6) in naturally-exposed volunteers and by 100% (1/1) of volunteers immunized with irradiated sporozoites.

RF scores show that 28.6% (2/7) to 100% (1/1) of subjects from a malaria-endemic area responded to **GIAGGLALL** (60, 63, 101) which is located near the C-terminus. **LLACAGLAYK** was detected in two different study populations: in naturally-exposed volunteers [RF = 22.2% (2/9)] and in study participants who had been experimentally immunized with irradiated sporozoites [75% (3/4)] (74, 145). **LLACAGLAYK** was also detected by malaria-naïve subjects immunized with TRAP [5.3% (1/19)].

## Apical Membrane Antigen 1 (AMA1)

AMA1 is both blood stage as well as a sporozoite stage antigen (22). It is important for invasion of erythrocytes, which makes AMA1 a target used as blood stage vaccine candidate (22, 188–191). Invasion of erythrocytes is a fast and complex process that takes a series of distinct steps. Firstly, merozoites, the invasive form of *Plasmodia*, attach to the host cell. Secondly, reorientation of the merozoites brings the apical end of the parasite toward the host cell. Finally, the erythrocytes are invaded (192–194).

In order to enter red blood cells (RBC), merozoites release specialized proteins out of secretory organelles called micronemes and rhoptries. AMA1 is one of the proteins, that are released to the surface of merozoites prior to invasion (188, 189, 195). A tight junction between merozoites and RBC is formed with an AMA1 and Rhoptry neck protein complex (RON) (190). The RON protein complex is initially stored within the rhoptries and then inserted into the RBC membrane (22). This junction then allows invagination of the host cell (191).

AMA1 is a 622 AA long protein. At its N-terminus, AMA1 consists of a propeptide that is removed before translocation of the protein onto the merozoite's surface (Figure 3A). This cleavage turns the precursor protein (83 kDa) into the mature 62-kDa form. The mature form has structural features of a type 1 integral membrane protein (188, 189, 195).

The ectodomain consists of three domains that is the extracellular part of AMA1 (196). Domain I and II show a high level of sequence conservation across *Apicomplexa*, suggesting a conserved function for these domains, whereas domain III is divergent (197). Domain I and II contain a PAN fold, a flexible loop structure with disulfide bonds. These two domains fold together and form a large hydrophobic cleft on the surface of AMA1 (198). This cleft is speculated to be a ligand-binding pocket (198). It is likely that the flexible and polymorphic loops protect the conserved hydrophobic cleft from detection by the host immune system (197).

All published epitopes on AMA1 were discovered in studies with malaria-naïve vaccines (Figure 3B).

The majority of epitopes has been described and published by Sedegah et al. (172). The malaria-naïve study population was immunized with the NMRC-M3V-Ad-PfCA vaccine, which is a combination of two separate recombinant adenovirus 5 constructs, one expressing *P. falciparum* CSP and the other expressing full-length *P. falciparum* AMA1 (both strain 3D7). In this study, the *P. falciparum*-specific CD8+ T cells were detected by ELISPOT. Five subjects were tested, and the response frequencies varied from 20% (1/5) to 40% (2/5). **TLDEMRHFY** and **NEVVVKEEY** are the only CD8+ T cell epitopes that were detected with tetramer-technology after immunization with the NMRC-M3V-Ad-PfCA vaccine (120). **TLDEMRHFY** had a response frequency of 66.7% (2/3) and **NEVVVKEEY** of 100% (1/1). Other epitopes were detected after immunization with DNA plasmids and adeno-viral vectors of CSP and AMA1 (117, 178).

## Liver Stage Antigen 1 and 3 (LSA1, LSA3)

The function of liver stage antigen 1 (LSA1) in *P. falciparum* malaria is not clear. The protein, which consists of 1,162 AA (Strain: 3D7) (199), is only expressed during the liver stage and detectable in the parasitophorous vacuole (200). The potential importance of LSA1 as a vaccine candidate was suggested by the observation that the epitope **KPIVQYDNF** restricted by HLA-B53 is associated with resistance to severe malaria (84) (Figure 4). RF varied from 23.1% (3/13) to 50% (3/6) in study participants who were naturally exposed to malaria (23, 63, 70, 100–102).

Two epitopes restricted by the protective HLA-B53 alleles have also been found in LSA3 (Figure 5): **EPKDEIVEV** (103) and **APFISAVAA** (102). Response frequency of **EPKDEIVEV** was 16.7% (1/6) and **APFISAVAA** showed a RF of 28.6% (2/7). Both epitopes were detected in a naturally exposed study population.

## Circumsporozoite-Related Protein (EXP1)

Inside the host cell, malaria parasites mature within the parasitophorous vacuole (PV) separated from the cytosol by the PV membrane (PVM) (201). The PVM serves as an interface between parasite and host with important functions like nutrient acquisition, host-cell remodeling, waste disposal, environmental sensing, and protection of the parasite (202). Within the PVM, the circumsporozoite-related protein (EXP1) is a component that is highly expressed during the blood and liver stage (203). Although this protein is likely to be essential, its exact function in the *P. falciparum* malaria life cycle is far from clear. EXP1 is transported out of the parasite into the parasitophorous vacuole membrane (204, 205) after invasion of host cells (206). It is then integrated in the PVM with the C-terminus exposed to the host cell cytosol and the N-terminus in the lumen of the PVM (207). EXP1 has been described to act as a glutathione S-transferase (GST) that protects the parasites from oxidative stress (208) and its enzymatic activity is inhibited by artesunate, a frontline antimalarial drug (208). EXP1 seems also to be essential for asexual development and the progression across the RBC cycle because the gene is one of the most abundantly transcribed loci during the ring and early trophozoite stages. This is the asexual growth phase inside erythrocytes (209, 210).

EXP-1 is a 162 AA long protein consisting out of a signal sequence (AA 1–23), an N-terminus (AA 23–79), a transmembrane domain (AA 79–101), and a C-terminus (AA 101–162) (211). Six epitopes within this protein have been discovered (Figure 6).

All epitopes were detected in subjects from an endemic area. Interestingly, no epitope within the N- or C-terminus has been described so far.

## Merozoite Surface Protein 1 (MSP1)

In the blood phase, the invasion of human erythrocytes is mediated through different proteins. Merozoite surface proteins (MSPs) are thought to be the primary ligands responsible for the low-affinity interactions between the merozoites and the erythrocytes [reviewed in Cowman and Crabb (193)]. The most abundant MSP is the glycosylphosphatidylinositol (GPI)-anchored MSP1 (212). Merozoite surface proteins present themselves as promising vaccine candidates because of their location on the parasite surface and thus exposure to the host immune system. It has been demonstrated that antibodies against MSPs are able to decrease parasitemia *in vivo* (213, 214). Within MSP1, which consists of 1,720 AA (Sequence 3D7) (199), only one CD8+ T cell epitope has been described so far (Figure 7): **KLKEFIPKV** is restricted by HLA-A^*^02:01 and was detected via secreted MHC/mass spectrometry ligand presentation in monochain transgenic mice (143).

## Sexual Stage-Specific Protein (Pfs16)

A small proportion of the asexual parasite population enters the sexual pathway and develops into male and female sexual forms called gametocytes, which are essential for transmission to the mosquito vector (215). These gametocytes are inside the parasitophorous vacuole membrane (PVM) and the sexual stage-specific protein (Pfs16), which is 157 AA in length (strain NF54) (199), is part of that membrane and also required for gametocyte maturation (216–219). So far, only one CD8+ T cell epitope has been detected within Pfs16 (Figure 7). **MPLETQLAI** had a response frequency of 100% (4/4) in subjects immunized with irradiated sporozoites. It was also tested in volunteers from a malaria endemic area [RF: 6.7% (3/45)−30% (3/10)] (69, 74).

## Sporozoite Threonine and Asparagine-Rich Protein (STARP)

Sporozoite threonine and asparagine-rich protein (STARP) is expressed on the surface of sporozoites that invade hepatic cells, which suggests that it plays a role during parasite entry into the hepatic cell and infection (220). The STARP protein, which consists of 594 AA (strain: 3D7) (199), has been considered to be a potential pre-erythrocytic vaccine candidate because antibodies to this antigen could block *P. falciparum* sporozoite invasion of hepatocytes (221). So far, only one epitope has been located within STARP (Figure 7). **MINAYLDKL** is restricted by HLA-A^*^02:01 and ^*^02:02. All studies that were conducted included subjects from endemic areas. Response frequency varied from 11.8% (2/17) to 75% (6/8) (60, 101, 141).

## Discussion

Here we present a comprehensive overview of all human *P. falciparum*-specific CD8+ T cell epitopes published so far, and show their role in the Plasmodium life cycle, potential importance for vaccine development, point out areas where there are still knowledge gaps and outline future directions.

We summarize the data of 132 different *P. falciparum*-specific CD8+ T cell epitopes that have been described in the literature, the respective study population and response frequency, methodology used for detection, and corresponding restricting HLA-molecule.

We hope, that this review will be used as a tool for the selection of suitable epitopes for MHC tetramer synthesis in order to investigate the phenotype of *P. falciparum*-specific CD8+ T cells, for immunomonitoring in vaccine trials, and finally to assist researcher interested in the development of a subunit vaccine. The different and detailed epitope maps give an overview of all epitopes within the antigens (Figures 1–7). This shows the immunodominant areas of a protein as well as regions that do not elicit a CD8+ T cell immune response.

Notably, the majority of the described *P. falciparum*-specific CD8+ T cell epitopes were discovered in human vaccine trials conducted in malaria-naïve volunteers (121, 145, 172). It has to be taken into account that vaccination with only one *Plasmodium* strain [namely strain 3D7 and NF54 (137, 175, 222)] does not represent a natural malaria infection which can be caused by several strains which might affect the priming and shaping of the T cell response (93, 223). Single-strain vaccines against intense heterogeneous parasite exposure are most likely not going to be successful (15). Further vaccine trials should target more than one antigen from different life-cycle stages to elicit broader immune responses (15, 178). Since to date only a few malaria antigens have been used in vaccine studies, epitopes located within these specific regions are overrepresented in the literature. This demonstrates that the choice of antigens in vaccine trials influences our knowledge about epitopes, but our knowledge about epitopes should also be considered for future vaccine designs. Next to eliciting stronger immune responses, it is widely believed that future malaria vaccines should include a larger number of epitopes in order to show broader protection (15).

In most studies summarized in this review, only small and partly pre-selected (e.g. for HLA-A^*^2) study populations were tested (an average of 10.3 subjects). Notably, the response frequencies varied between the different study populations. The group that was immunized with irradiated sporozoites had the highest response frequency (72.6%). The group that was immunized with parts of the plasmodial antigens showed a response frequency of 26.8% and the volunteers from an endemic area had a RF of 16.0% only. The very high RF after immunization with irradiated sporozoites might explain why this is the most effective form of vaccination so far (15).

Only few studies used comprehensive, overlapping peptide libraries for the few antigens tested and mostly already known or *in silico* predicted peptides were utilized (106, 222). Many studies worked with peptide pools and single peptides could not be identified (178, 222). A number of studies focused on HLA-A^*^2 restricted epitopes, which is the most common expressed Caucasian HLA molecule but not that common in Africa (141, 146). Epitopes recognized in the context of a more diverse set of HLA molecules need to be defined (110).

Furthermore, there is a need for new vaccine targets because most of the known targets have been the same over the last decades (224). For example, only one CD8+ T cell epitope has been defined for an antigen from the sexual stage (Pfs16) (which could potentially prevent transmission by the mosquito) (74). Hill et al. suggest that the search for an association between the ability to respond to a particular malaria antigen or epitope with protective HLA types could be useful to identify an antigen that confers naturally acquired immunity and use it as an antigen for vaccine trials (84).

Indeed, the missing knowledge (and attention paid to) the discovery of new specific malaria epitopes combined with the overall low frequency of *P. falciparum*-specific T cells in the peripheral blood has led to the fact that most immunological studies rather analyzed the function and phenotype of bulk CD8+ T cells (225, 226). Studies on the phenotype and function of *P. falciparum*-specific T cells are largely missing. Fine mapping of the optimal length of the CD8+ T cell epitopes and investigation of their HLA restriction will allow the synthesis of suitable MHC tetramers for phenotypical analysis, most likely by employing further *in vitro* column tetramer-enrichment techniques (227).

The majority of studies on the breadth and specificity of the *P. falciparum*-specific T cell answer was published before 2000 (60, 63, 159). For this and other reasons only few studies used novel and sensitive technologies like ICS, MHC tetramer or even ELISPOT assays (117, 119, 137). Indeed, the last experimental mapping paper was published in 2016 (117, 119). This mirrors the fact that the most intense immunological investigations in the field of malaria epitope discovery took place in the late-1980s and 1990s (110).

Apart from the multicolor flow ICS analysis of vaccine induced *P. falciparum*-specific responses in a couple of vaccine studies, few studies have looked at other effector cytokines or the degree of multifunctionality of antiparasitical cytokines in malaria patients e.g., using multicolor flow cytometry based ICS (228, 229).

As future direction (Table 2), mass spectrometry studies e.g., of dendritic cells exposed to malaria parasites, or hepatocytes infected with liver stage antigen in order to identify further protective epitopes will potentially be another highly useful tool for epitope discovery (231).

**Table 2:** Future directions.

(1) Employment of different *P. falciparum* protein based comprehensive overlapping peptide sets to map the full breadth and specificity of the human malaria-specific CD8+ T cell repertoire.
•Use of sensitive technologies like ELISPOT, ICS.
•Experimental fine mapping of the optimal length and HLA restriction of malaria-specific CD8+ T cell epitopes.
•Screening of large cohorts of patients and vaccines with diverse HLA backgrounds.
•Investigation of the breadth and specificity of the T cell response primed and directed against novel, promising vaccine candidates (e.g., SPECT-1, PFL1620, MALP1.22, PF10925w, PF14_0051 (230).
•Use of “Next Generation Sequencing” for full analysis of the T cell repertoire of malaria patients.
•Construction of novel human MHC class I malaria multimers.
•Further development of mass spectrometry methodologies e.g., of DCs exposed to malaria parasites or hepatocytes infected with liver stages to identify protective epitopes.
(2) Multichannel *ex vivo* phenotypic and functional analysis of malaria-specific T cells using peptide pools, malaria-specific tetramers and employing novel, highly sensitive assays.
(3) Sequencing of circulating *P. falciparum* genome to identify consensus sequences for different malaria antigens, to understand cross-strain immunoreactivity, examine immune pressure, immune escape, and cost of fitness.
(4) Investigation of tissue-resident malaria-specific T cells (e.g., via fine-needle aspiration in the liver).
(5) In iteration based on (1–4) construction and testing of novel multiepitope vaccine constructs containing larger number of antigenic regions.

Interestingly, many of the described *P. falciparum*-specific T cell responses are situated in structurally and functionally important regions. For example: the epitopes that have been described for EXP1, are mostly located within the transmembrane domain but not in the C- nor N-terminus (74, 145). Another example is the epitope **LRKPKHKKL** within the CSP (63, 121, 145) that overlaps with region I, a region which is conserved in almost all *Plasmodium* species and has an important function for hepatocyte invasion (152). Analysis of these and further interactions might be important to assess the role of T cell escape and fitness cost of certain epitopes for future vaccine design.

Supplementary Figures 1–4 depict the entropy blots of the proteins CSP, TRAP, AMA1, and EXP1. This Shannon Entropy-one calculation by the HIV Sequence database compares different sequences of the proteins found on Uniprot, altogether demonstrating the high diversity of the different malaria antigens and strains (199) but also showing the lack of antigen sequencing. For example, only 12 different sequences could be found in open resources for the *P. falciparum* antigen EXP1 (199). Many regions within these antigens used for the entropy map are thought to have important physiological functions but, nevertheless, are polymorph. *P. falciparum*-specific peptides made with a consensus sequence selected out of the sequence of different *P. falciparum* strains could be used for future studies, ideally such a consensus sequence should be designed specifically for individual geographic areas. Studies that investigate the specific CD8+ T cell response should also test different variants from different *Plasmodium* strains if possible. González et al. e.g., tested the variants **TLRKPKHKKL** and **KLRKPKHKKL**, both restricted to HLA-A^*^02:01, in 17 subjects (141). RF for **TLRKPKHKKL** was 5.9% whereas for **KLRKPKHKKL** it was 17.6%. This demonstrates that small changes within the AA structure of an epitope can change the binding behavior and improve the priming of CD8+ T cells in context of vaccine design.

Most studies were performed either in the setting of a protective vaccine or in healthy exposed volunteers. Future studies should look at the kinetics of the priming of the CD8+ T cell response during a natural and acute malaria infection with longitudinal long-term follow up to understand the longevity of the *P. falciparum*-specific T cell response. These studies will be crucial to evaluate the difference between vaccine and naturally induced *P. falciparum*-specific T cell responses. Epitope data in the context of clinical stages (age, parasitaemia, complicated, or uncomplicated course of disease, etc.) during an acute disease are lacking because specific CD8+ T cells have rarely been investigated in malaria patients.

Even though a T cell response is mounted against a certain epitope does not mean that this response will correlate with protection. E.g., RAS-vaccines are likely to induce CD8+ T cell responses to many sporozoite proteins, but only a subset of these will be presented by liver stage infected hepatocytes which are the target of protective immunity (232). In different murine and human immunological studies, it could also be demonstrated that only certain epitopes correlate with protection (233–235). The identification of further protective epitopes is therefore necessary. At the same time any future malaria vaccine will have to render a broad population-wide coverage since a protective epitope might be presented by only certain HLA molecules.

Additionally, studies looking at the breadth, specificity, and functionality of tissue-resident, *P. falciparum*-specific T cells in humans are missing. Fine needle aspiration could be introduced as a useful tool to investigate liver-resident T cells because understanding of the CD8+ T cell response from peripheral blood samples has proven to be difficult (236).

The biggest effort in vaccine development was put into the assessment of the humoral response (237). Nevertheless, has this approach not lead to an effective vaccine (e.g., the RTS,S vaccine). The cellular response should therefore not be disregarded (8). On that note, it has become clear that a strong and long lasting CD8+ T cell response is dependent on CD4+ T cells (238, 239). The *P. falciparum*-specific CD4+ T cell response has so far been investigated in more detail with 596 epitopes published compared to the 147 published CD8+ T cell epitopes. This may simply reflect the technical ease of certain assays (lymphoproliferation for CD4+ T cell assessment compared to cytotoxicity assays for CD8+ T cells) but it could also reflect the focus of scientific investigations (110). It is also possible that this disparity is due to biological difficulties in the detection of *P. falciparum*-specific CD8+ T cells (as discussed above).

Further and more comprehensive studies using different cohorts, novel and more sensitive immunological techniques and assays and comprehensive, overlapping peptide sets for a number of different malaria proteins are warranted. In other words: the lacking knowledge of potential *P. falciparum*-specific CD8+ T cell epitopes is hampering optimization for a malaria vaccine.

In summary, the discovery of the full breadth and exact specificities of the *P. falciparum*-specific immune response on an epitope level is of utmost importance for the detailed understanding of the role of CD8+ T cells for control of disease and will give us the tools to optimize vaccine design, immune monitoring of future malaria vaccine trials and to better understand naturally acquired immunity.

## Data Availability

All datasets generated for this study are included in the manuscript and/or the supplementary files.

## Author Contributions

JH, TJ, and JSzW: conception. JH and JSzW: first draft. All authors: important contributions and proofread.

### Conflict of Interest Statement

The authors declare that the research was conducted in the absence of any commercial or financial relationships that could be construed as a potential conflict of interest.

## Footnotes

^1^*PubMed Help* [Internet]. Bethesda, MD: National Center for Biotechnology Information (US); 2005-. Available online at: https://www.ncbi.nlm.nih.gov/books/NBK3827/. Home - PubMed – NCBI; https://www.ncbi.nlm.nih.gov/pubmed/. (Accessed: August 29, 2018).

## Supplementary Material

Supplementary Table 1 *P. falciparum* CD8+ T cell epitopes described in humans. All *P. falciparum*-specific CD8+ T cell epitopes are listed in this table. The reference number (1–35) refers to the number written in each epitope box on the epitope maps. The antigen, the study population, the method used, the response frequency as well as the allele name responding to a certain epitope can be found in this table. Response frequency (RF) represents the number of respondents over the number of subjects tested, when provided by the investigator for the respective assay. ND stands for “not determined” when the investigator did not share this information in the article.

Supplementary Figure 1 Entropy map of *P. falciparum* CSP. The entropy value was calculated for each AA position. For this calculation 100 different *P. falciparum* sequences were compared. The first 78 aminoacids are well conserved within the N-terminus. The repeat region differs in the number of NANP repeats. The C-terminus is very entropy-rich. We chose the first 100 sequences available on UniProt for this Shannon Entropy-one calculation by the HIV Sequence database.

Supplementary Figure 2 Entropy map of TRAP. The entropy value was calculated for each AA position. For this calculation six different *P. falciparum* sequences were compared. We chose six sequences available on UniProt for this Shannon Entropy-one calculation by the HIV Sequence database.

Supplementary Figure 3 Entropy map of *P. falciparum* AMA1. The entropy value was calculated for each AA position. For this calculation 680 different *P. falciparum* sequences of AMA1 were compared. We chose all sequences available on UniProt for this Shannon Entropy-one calculation by the HIV Sequence database.

Supplementary Figure 4 Entropy map of *P. falciparum* EXP1. The entropy value was calculated for each AA position. For this calculation 12 different *P. falciparum* sequences of EXP1 were compared. We chose all sequences available on UniProt for this Shannon Entropy-one calculation by the HIVSequence database.

## Abbreviations

AA: amino acids
AMA1: apical membrane antigen
APC: antigen presenting cells
CSP: circumsporozoite protein
CTL: cytotoxic T lymphocytes
ELISA: enzyme linked immunosorbent assay
EXP1: exported protein one
GLURP: glutamate-rich protein
HSPGs: heparan sulfate proteoglycans
HLA: human leukocyte antigens
ICS: intracellular staining
IEDB: The Immune Epitope Database and Analysis Resource
IFNγ: interferon gamma
Il: interleukin
LSA: liver stage antigen
MHC: major histocompatibility complex
MSP: merozoite surface protein
P: Plasmodium
Pf: Plasmodium falciparum
PfEMP: erythrocyte membrane protein
Pfs16: sexual stage specific protein
RF: response frequency
STARP: sporozoite threonine and asparagine-rich protein
TNFα: tumornecrosis factor alpha
TRAP: thrombospondin-related antigen.
