# Supplementary material for: Comprehensive Review of Human Plasmodium falciparum-Specific CD8+ T Cell Epitopes
Source: Front Immunol. 2019 Mar 21;10:397. doi: 10.3389/fimmu.2019.00397 (PMC6438266; doi:10.3389/fimmu.2019.00397)
Supplement: Supplementary Figure 1 — Entropy map of P. falciparum CSP. The entropy value was calculated for each AA position. For this calculation 100 different P. falciparum sequences were compared. The first 78 aminoacids are well conserved within the N-terminus. The repeat region differs in the number of NANP repeats. The C-terminus is very entropy-rich. We chose the first 100 sequences available on UniProt for this Shannon Entropy-one calculation by the HIV Sequence database. [file Data_Sheet_1.PDF]

Supplementary figures:

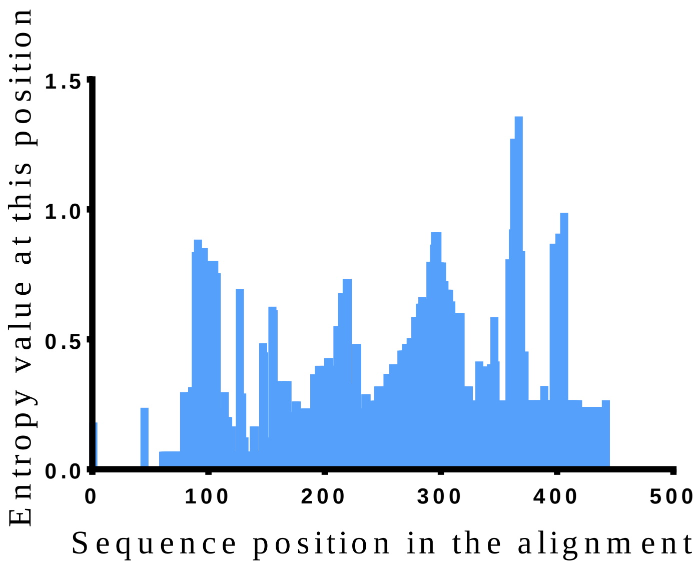

Supplementary figure 1. Entropy map of *P. falciparum* CSP. The entropy value was calculated for each AA position. For this calculation 100 different *P. falciparum* sequences were compared. The first 78 aminoacids are well conserved within the N-terminus. The repeat region differs in the number of NANP repeats. The C-terminus is very entropy-rich. We chose the first 100 sequences available on UniProt for this Shannon Entropy-one calculation by the HIV Sequence database.

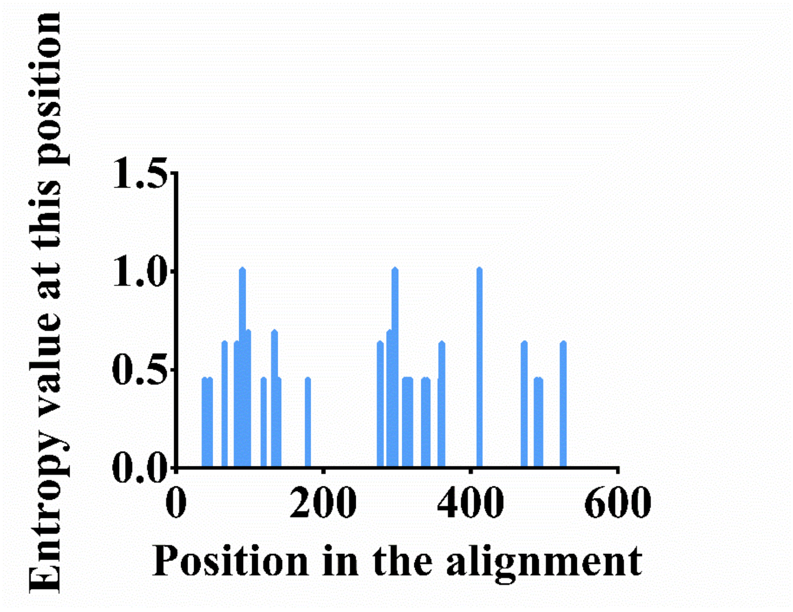

Supplementary Figure 2. Entropy map of TRAP. The entropy value was calculated for each AA position. For this calculation 6 different *P. falciparum* sequences were compared. We chose 6 sequences available on UniProt for this Shannon Entropy-one calculation by the HIV Sequence database.

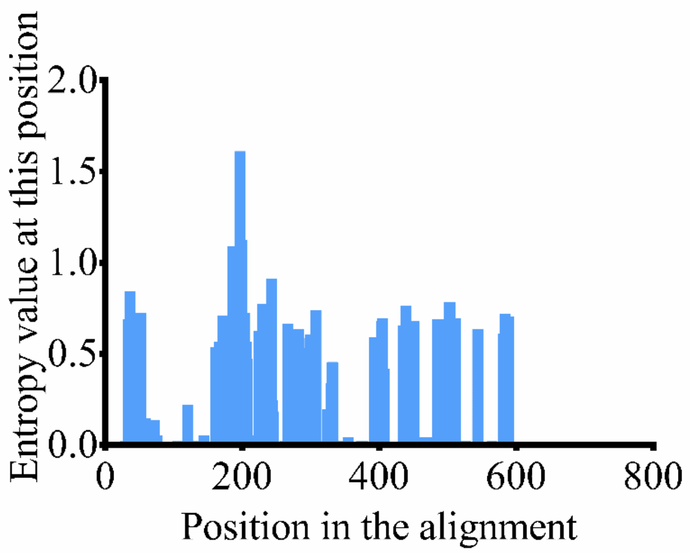

Supplementary Figure 3. Entropy map of *P. falciparum* AMA1. The entropy value was calculated for each AA position. For this calculation 680 different *P. falciparum* sequences of AMA1 were compared. We chose all sequences available on UniProt for this Shannon Entropy-one calculation by the HIV Sequence database.

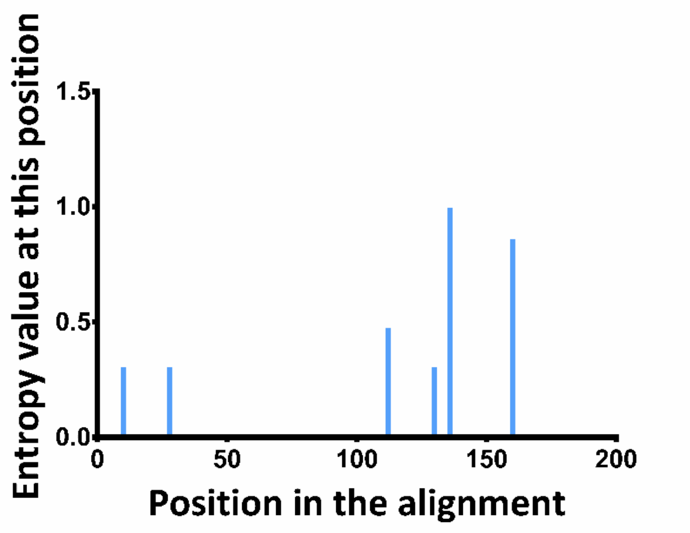

Supplementary Figure 4. Entropy map of *P. falciparum* EXP1. The entropy value was calculated for each AA position. For this calculation X different *P. falciparum* sequences of EXP1 were compared. We chose all sequences available on UniProt for this Shannon Entropy-one calculation by the HIV Sequence database.
